# Reliability and feasibility of visual grading systems and quantitative indexes on [^99m^Tc]Tc-DPD imaging for cardiac amyloidosis

**DOI:** 10.1038/s41598-022-21603-8

**Published:** 2022-10-14

**Authors:** Sungwoo Bae, Joonhyung Gil, Jin Chul Paeng, Eun-Ah Park, Seung-Pyo Lee, Hongyoon Choi, Keon Wook Kang, Gi Jeong Cheon, Dong Soo Lee

**Affiliations:** 1 Department of Molecular Medicine and Biopharmaceutical Sciences, Graduate School of Convergence Science and Technology, Seoul National University, Seoul, Korea; 2 Department of Nuclear Medicine, Seoul National University College of Medicine, 103 Daehak-ro, Jongno-gu, Seoul, 03080 Republic of Korea; 3 Department of Radiology, Seoul National University Hospital, Seoul, Korea; 4 Department of Internal Medicine, Seoul National University Hospital, Seoul, Korea; 5 Institute of Radiation Medicine, Medical Research Center, Seoul National University, Seoul, Republic of Korea

**Keywords:** Cardiology, Cardiovascular diseases, Molecular medicine

## Abstract

We aimed to evaluate the reliability and feasibility of visual grading systems and various quantitative indexes of [^99m^Tc]Tc-DPD imaging for cardiac amyloidosis (CA). Patients who underwent [^99m^Tc]Tc-DPD imaging with suspicion of CA were enrolled. On the planar image, myocardial uptake was visually graded using Perugini’s and Dorbala’s methods (PS and DS). As [^99m^Tc]Tc-DPD indexes, heart-to-whole body ratio (H/WB) and heart-to-contralateral lung ratio (H/CL) were measured on planar image. SUVmax, SUVmean, total myocardial uptake (TMU), and C-index were measured on SPECT/CT. Inter-observer agreement of the indexes and their association with visual grading and clinical factors were evaluated. A total of 152 [^99m^Tc]Tc-DPD images, of which 18 were positive, were analyzed. Inter-observer agreement was high for both DS (κ = 0.95) and PS (κ = 0.96). However, DS showed a higher correlation with quantitative indexes than PS. Inter-observer agreement was also high for SPECT/CT indexes, particularly SUVmax. SUVmax was significantly different between different DS groups (*P* = 0.014–0.036), and showed excellent correlations with H/WB and H/CL (*r* = 0.898 and 0.910). SUVmax also showed significant differences between normal, AL, and ATTR pathology (*P* = 0.022–0.037), and a significant correlation with extracellular volume on cardiac MRI (*r* = 0.772, *P* < 0.001). DS is a visual grading system for CA that is more significantly matched with quantitative indexes than PS. SUVmax is a reliable quantitative index on SPECT/CT, with a high inter-observer agreement, correlations with the visual grade, and potential association with cardiac MRI findings.

## Introduction

Cardiac amyloidosis (CA) is a fatal manifestation of systemic amyloidosis that leads to diastolic dysfunction of left ventricle (LV) and heart failure. Immunoglobulin light chain amyloidosis (AL) and transthyretin amyloidosis (ATTR) are most common types of CA. Despite the clinical importance of CA, many patients are misdiagnosed and found in advanced stages of the disease^1,2^. Recent progress in the treatment of CA has triggered the need for early and precise diagnosis of CA, as early treatment can prevent disease progression^3^. Although endomyocardial biopsy (EMB) provides a definitive diagnosis, it is invasive and occasionally causes severe complications^4^. In this regard, imaging modalities have been investigated for diagnosis of CA^1^. Currently, bone scan using [^99m^Tc]Tc-3,3-diphosphono-1,2-propanodicarboxylic acid (DPD) or [^99m^Tc]Tc-pyrophosphate (PYP) is deemed one of standard diagnostic procedures for CA with high accuracy^5^. For interpretation of bone scan findings in CA, some visual grading systems and quantitative indexes have been proposed.

Single-photon emission computed tomography (SPECT)/computed tomography (CT) has advantages over planar imaging, like accurate anatomical localization and quantification. It has been reported in CA that quantitative indexes on [^99m^Tc]Tc-DPD SPECT/CT are well matched with visual grades^6,7^, superior to planar image indexes^7^, and well correlated with other radiological measures of amyloid burden^7,8^ or cardiac function^9^. Quantitative indexes on [^99m^Tc]Tc-PYP SPECT/CT also showed similar results and presented high intra-observer agreement^10,11^. However, there is still no definite consensus on the optimal use of visual grading system on bone scan or quantitative indexes on SPECT/CT.

In this study, we evaluated the reliability of the two well-known visual grading systems for bone scan in CA, in terms of inter-observer agreement and correlation with other quantitative indexes. Also, several quantitative indexes on SPECT/CT that have been proposed for CA were also tested for their reliability and feasibility.

## Results

### Patients

A total of 149 patients were retrospectively enrolled, and 152 cases of [^99m^Tc]Tc-DPD images were analyzed because three patients underwent imaging study twice. The characteristics of 149 patients are summarized in Table 1. Among the 152 images, 18 showed positive findings with visual grades ≥ 1. EMB was performed in 27 patients for whom the definitive diagnosis of CA or an exclusion was of a clinical interest; 13 were diagnosed with CA and 14 were normal. Correlations between [^99m^Tc]Tc-DPD image findings and clinical characteristics were analyzed in 142 patients, with excluding seven patients who were finally confirmed as ischemic cardiomyopathy. Echocardiography was performed in 124, of which SUVmax was available in 59 cases. The median time interval from [^99m^Tc]Tc-DPD imaging was 8.5 days (range 0–344 days). Cardiac MRI was performed in 30, of which 20 cases had ECV and SUVmax measurements. The median time interval between [^99m^Tc]Tc-DPD imaging and MRI was 3.5 days (range 0–339 days).

**Table 1:** Characteristics of enrolled patients.

Characteristics	Values
Age, years (range)	78 (28–93)
**Gender**	
Male	69 (46.3%)
Female	80 (53.7%)
**DS**	
0	131 (87.9%)
1	5 (3.4%)
2	3 (2.0%)
3	10 (6.7%)
**PS**	
0	131 (87.9%)
1	5 (3.4%)
2	6 (4.0%)
3	7 (4.7%)
**Pathology (n = 27)**	
Normal	14 (51.9%)
AL	4 (14.8%)
ATTR	9 (33.3%)

*ATTR* transthyretin amyloidosis, *AL* light-chain amyloidosis, *DS* Dorbala grading system, *PS* Perugini grading system.

### Planar image indexes: inter-observer agreement and correlation with visual grade

Inter-observer agreement between the two independent readers across 152 images was excellent for both visual grades, DS (κ = 0.95, 95% CI 0.89–1.00) and PS (κ = 0.96, 95% CI 0.91–1.00) (Supplementary Table 1). Quantitative indexes on planar image across 18 positive cases also showed high ICC between the two readers (0.918 for H/WB and 0.901 for H/CL, Supplementary Table 2). On the Bland–Altman plot, mean ± 1.96 SD values of the differences were 0.01 ± 0.02 and − 0.39 ± 1.47 for H/WB and H/CL, respectively (Supplementary Fig. 1).

**Figure 1:**
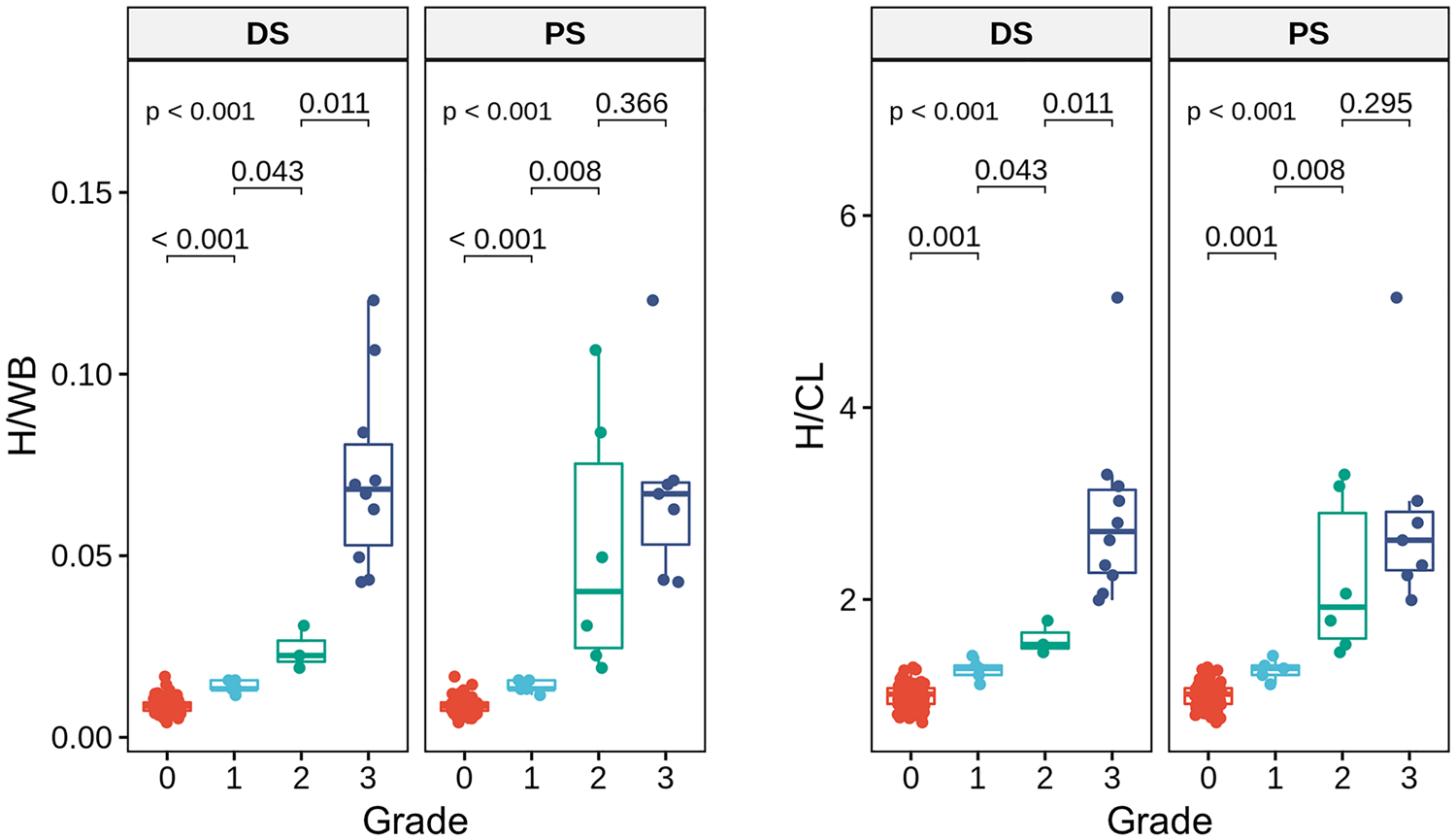
Planar image indexes according to the visual grades. H/WB and H/CL were significantly different between grades 0/1 and 1/2 for both DS and PS. However, H/WB and H/CL were significantly different between grades 2/3 only for DS, but not for PS.

H/WB and H/CL showed high correlations with visual grades. There were significant differences in H/WB and H/CL between grades 0 and 1, and between 1 and 2, for both DS and PS (*P* < 0.001 for both, Table 2). However, H/WB and H/CL were significantly different between grades 2 and 3 only for DS, but not for PS (Fig. 1). According to these results, DS was adopted as the visual grading system in the further analyses.

**Table 2:** Quantitative indexes according to the visual grades.

Indexes	Visual grade				*P*
	0	1	2	3	
**Planar indexes vs. DS**					
H/WB	0.01 ± 0.00	0.01 ± 0.00*	0.02 ± 0.01*	0.07 ± 0.05*	< 0.001
H/CL	0.99 ± 0.22	1.27 ± 0.22*	1.59 ± 0.34*	2.87 ± 1.80*	< 0.001
**Planar indexes vs. PS**					
H/WB	0.01 ± 0.00	0.01 ± 0.00*	0.05 ± 0.07*	0.07 ± 0.05	< 0.001
H/CL	0.99 ± 0.22	1.27 ± 0.22*	2.22 ± 1.61*	2.88 ± 2.07	< 0.001
**SPECT/CT indexes vs. DS**					
SUVmax		2.50 ± 1.55	4.07 ± 0.35*	15.19 ± 8.04*	< 0.001
SUVmean		1.09 ± 0.69	1.66 ± 0.47	6.20 ± 4.46*	< 0.001
TMU		330 ± 168	413 ± 93	2263 ± 2204*	< 0.001
C-index		0.68 ± 1.25	1.21 ± 0.80	4.88 ± 1.63*	< 0.001

The quantitative indexes were represented as mean ± 1.96 SD.

*significantly different from the value of immediate lower grade.

### SPECT/CT indexes: inter-observer agreement and correlation with visual grade

**Figure 2:**
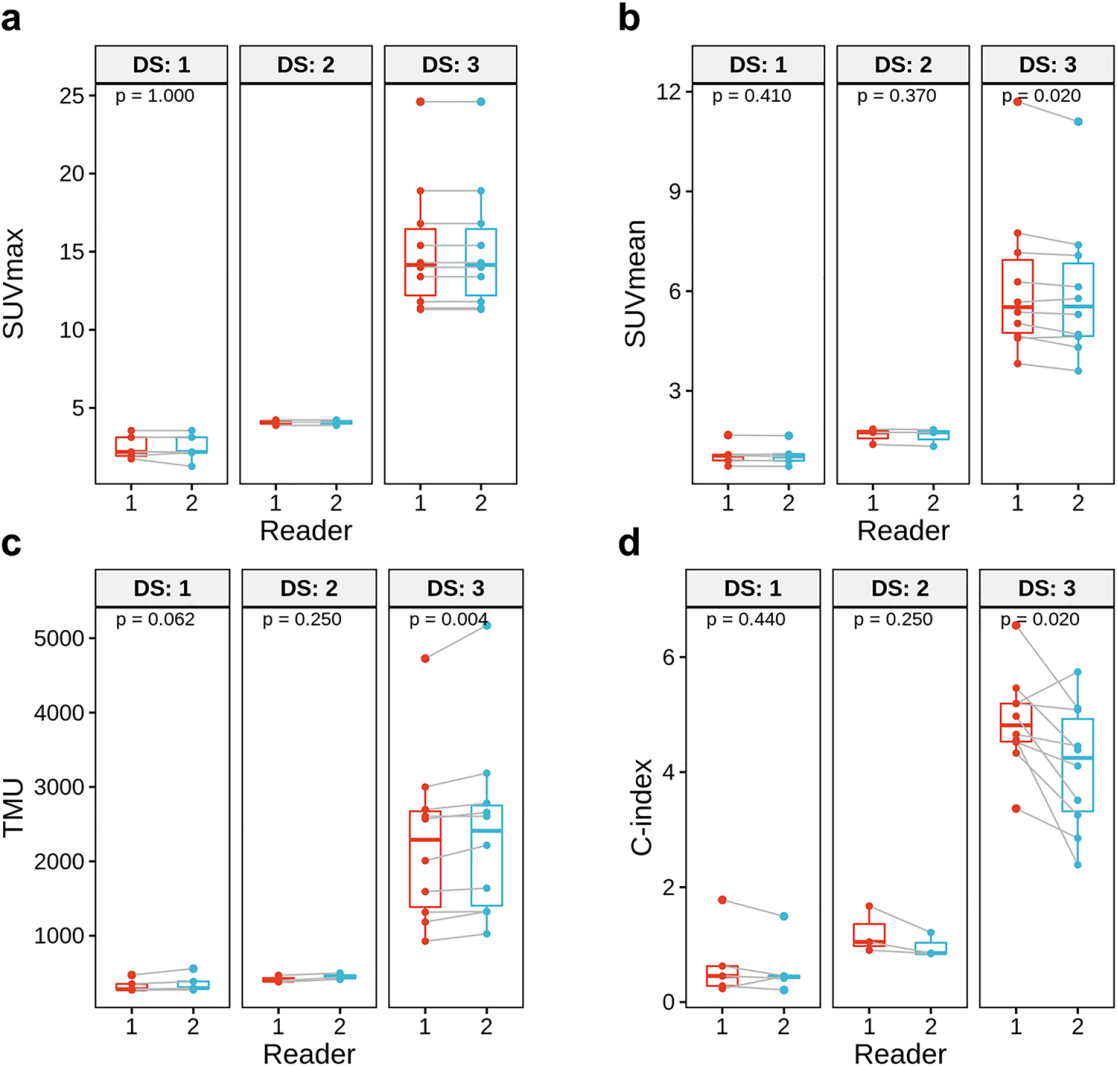
Inter-observer agreement of SPECT/CT indexes from the two readers in each visual grade group. SUVmax showed perfect agreement between the two readers in DS 2 and 3, despite a small difference in DS 1 (**a**), whereas other indexes showed some differences (**b–d**). In particular, C-index in DS 3 showed a significant difference between the two readers (**d**).

All SPECT/CT indexes showed high ICC between the two readers (0.958–1.000, Supplementary Table 2), which were higher than those of the planar indexes. Among them, SUVmax showed the highest ICC. When the agreement was analyzed in each visual grade group, SUVmax showed perfect agreement between the two readers in DS 2 and 3, despite a small difference in DS 1 (Fig. 2a). Other indexes also showed high ICCs between the two readers, although they were slightly lower than that of SUVmax (Supplementary Table 2, Fig. 2b–d). In particular, C-index in DS 3 showed a significant difference between the two readers (*P* = 0.020, Fig. 2d). On Bland–Altman plot, mean ± 1.96 SD values of the differences were 0.01 ± 0.25, 0.12 ± 0.36, − 87 ± 213, and 0.50 ± 1.34, for SUVmax, SUVmean, TMU, and C-index, respectively (Supplementary Fig. 1).

**Figure 3:**
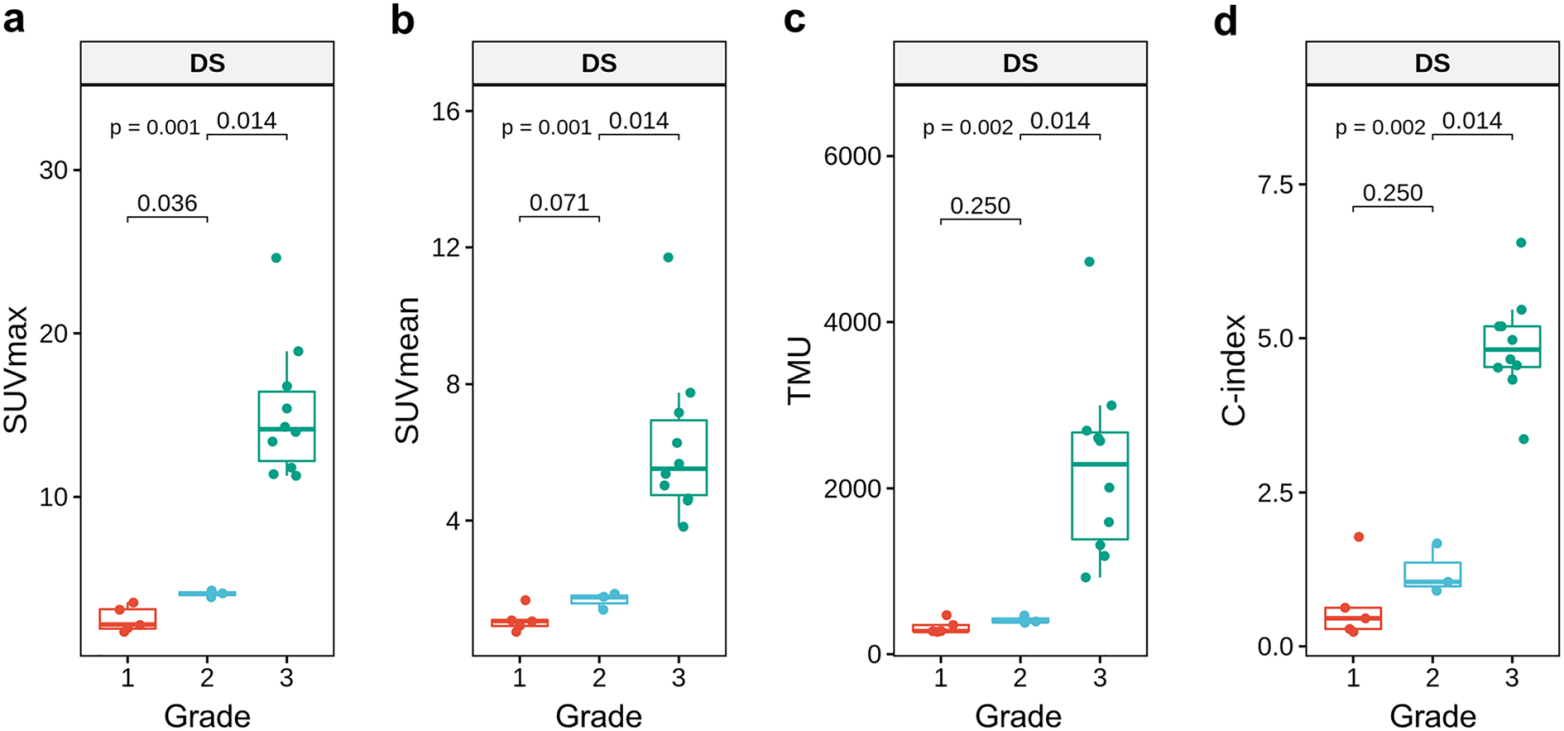
SPECT/CT indexes according to the visual grade, DS. SUVmax was significantly different between DS 1/2, and between 2/3 (**a**), whereas other indexes were not (**b–d**).

**Figure 4:**
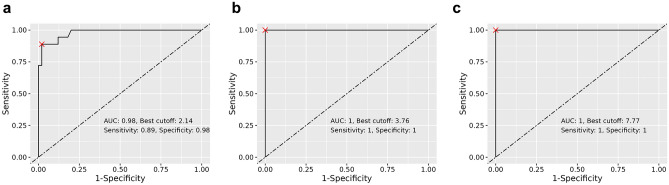
ROC analysis of SUVmax for differentiating the visual grade, DS. SUVmax values of 2.14, 3.76 and 7.77 were selected as the optimal cutoff values for differentiating DS 0/1 (**a**), 1/2 (**b**), and 2/3 (**c**), respectively.

SPECT/CT indexes showed associations with DS (Table 2), and SUVmax was significantly different between DS grades 1 and 2, and between 2 and 3, whereas other indexes were not (Fig. 3). SUVmax, SUVmean, and TMU showed excellent correlations with H/WB and H/CL (*r* = 0.898–0.974), whereas C-index showed a relatively lower correlation (*r* = 0.696 and 0.799) (Supplementary Fig. 2). In the ROC curve analyses in terms of SUVmax and DS, 2.14, 3.76 and 7.77 were selected as the optimal cutoff values for differentiating DS 0 vs. 1, 1 vs. 2, and 2 vs. 3, respectively (Fig. 4).

### Relation of quantitative indexes to pathologic diagnosis and clinical features

**Figure 5:**
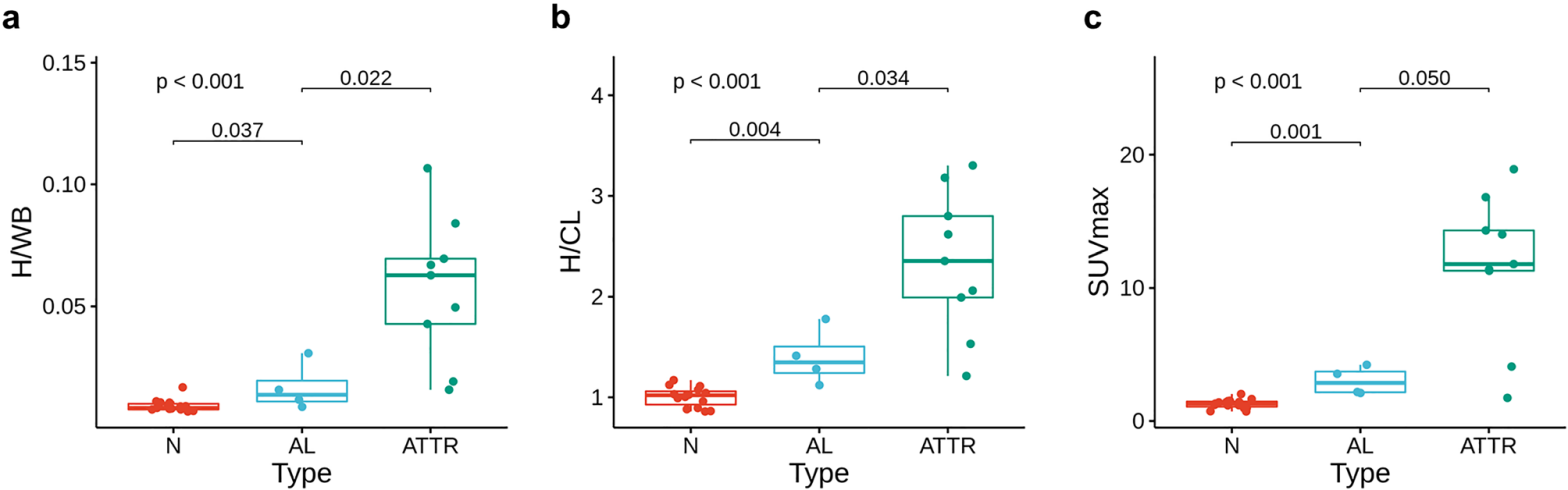
Quantitative indexes according to EMB results. H/WB, H/CL, and SUVmax were significantly higher in AL than in normal, and higher in ATTR than in AL.

In 27 patients who underwent EMB, H/WB, H/CL, and SUVmax were significantly different between patients diagnosed with normal, AL, and ATTR (Fig. 5). All the indexes were significantly higher in ATTR than normal and AL.

In 59 cases whose echocardiography and SUVmax data were available, E/A and E/e’ did not show significant correlation with DS, although there was a weak positive correlation between SUVmax and E/A (*r* = 0.268, *P* = 0.087) (Supplementary Fig. 3). In 20 cases whose cardiac MRI and SUVmax data were available, ECV was significantly different between DS 0 and 3 (*P* = 0.015) (Supplementary Fig. 4a), and ECV showed a significant correlation with SUVmax (*r* = 0.772, *P* < 0.001) (Supplementary Fig. 4b).

## Discussion

In this study, we evaluated visual grades and quantitative indexes of bone scan and SPECT/CT for CA, in terms of inter-observer agreement and correlation with other indexes. Both the two well-known visual grading systems showed high inter-observer agreement, and DS matched with H/CL and H/WB better than PS. All the tested SPECT/CT indexes showed superior inter-observer agreement to planar indexes. Particularly, SUVmax showed not only the highest inter-observer agreement but also significant correlations with other indexes.

Bone scan using [^99m^Tc]Tc-DPD or [^99m^Tc]Tc-PYP is an accurate and effective imaging for ATTR CA, although the exact mechanism of uptake is still unclear. Currently, the bone scan is included in the standard diagnostic processes recommended by major cardiology societies^12^. Because the uptake on bone scan is stronger in ATTR than in AL, visual grading of uptake have been proposed and grades 2 or higher are used for diagnostic criteria for ATTR CA in the recommendations. In the earlier proposed system, PS, attenuation of the bone uptake is evaluated in addition to myocardial uptake, whereas only the myocardial uptake compared to the rib uptake is considered in the DS. Although these visual grading systems are well established and have been used for clinical practice and many studies, they depend on expertise of readers and vulnerable to inter-observer variability. Thus, quantitative indexes have been proposed as complementary methods, and H/CL and H/WB are frequently used on planar scan^13^. As these indexes represent the degree of myocardial uptake, they are expected to match with DS better than PS. In our study, H/CL and H/WB showed clear differences between grades 2 and 3 in DS, but not in PS.

In recent years, quantitative SPECT/CT are used in clinical practice based on advances of scanners and imaging technology. The heart and reference organs can be accurately delineated on CT images, and SUV of a VOI can be measured by count corrections for attenuation, scatter, sensitivity, and others. There have been several quantitative indexes proposed for bone SPECT/CT in CA^6,7^. In the present study, all the tested SPECT/CT indexes showed higher inter-observer agreement than the planar image indexes, probably due to correct delineation of the heart based on CT images. Among them, SUVmax showed nearly perfect agreement between the two readers, except only two cases of grade 1 (Fig. 2a). It is a reasonable result because SUVmax is not affected by minor difference in segmentation if myocardial uptake is higher than adjacent tissues. The two cases that presented discrepant SUVmax between the two readers had mild uptake in the myocardium and spillover activity from the adjacent ribs may have affected the measurements. In contrast to SUVmax, C-index exhibited a relatively low inter-observer agreement and low correlations with planar indexes. As the spine uptake is used for calculation of C-index, it may be affected by abnormal spine uptake such as degenerative osteophytes.

In addition to inter-observer agreement, SUVmax showed high correlations with planar image indexes of H/WB and H/CL. SUVmean and TMU exhibited slightly higher correlation coefficients than SUVmax probably due to similarity of measuring methods. However, SUVmax was more effective to discriminate visual grades, particularly low grades 1 vs. 2 (Fig. 3) whereas other indexes were not. It is speculated that SUVmean and TMU underestimate myocardial uptake as they use the averaged SUV for the whole LV VOI. Additionally, spillover activity from the ribs to the LV cavity may affect the average uptake, especially in case of low-grade uptake.

In the ROC curve analyses, SUVmax showed excellent diagnostic performance (AUC 0.98–1.00) for discriminating visual grades. The optimal cutoff values of SUVmax for discriminating DS grades 0 vs. 1, 1 vs. 2, and 2 vs. 3 were 2.14, 3.76 and 7.77, respectively. In a recent study, SUVmax 6.0 was reported as an optimal cutoff value for discriminating grades 0/1 vs. 2/3^14^, which is different from ours. Thus, further studies are required for standardizing protocols for [^99m^Tc]Tc-DPD SPECT/CT acquisition and measurement.

SUVmax also showed significant differences according to the pathologic diagnosis, AL and ATTR, despite small case number of EMB. Although H/CL and H/WB were also significantly different according to the pathology, SUVmax showed lower variations in each group, compared to H/CL and H/WB (Fig. 5). SUVmax presented only a weak correlation with E/A, and E/e’ did not show significant correlation with any of the tested [^99m^Tc]Tc-DPD image indexes. In this study, many of the patients were negative for CA, and it is speculated that many patients with restrictive cardiomyopathy of other causes such as cardiac sarcoidosis and pulmonary hypertension have been included in the study^15,16^. However, ECV on cardiac MRI showed a significant correlation with SUVmax, which corresponds to previous reports^7,8^.

This study has some limitations. First, [^99m^Tc]Tc-DPD imaging is used as a screening test in our institution, the number of positive cases was small. Additionally, further studies of cardiac MRI and EMB were performed in a limited number of cases. However, it was not supposed to be a critical limitation for evaluating the inter-observer agreement and efficacy of SUVmax as a quantitative index for [^99m^Tc]Tc-DPD SPECT/CT. The sample size of 18 is acceptable in case the ICC is 0.90–0.97, and the statistical power and significance level is 0.8 and 0.05, respectively^17^. The varying time intervals (2–3 h) between [^99m^Tc]Tc-DPD injection and image acquisition would be another limitation. However, a previous study reported that interobserver agreement of visual grades and the H/CL index were comparable between 1 and 3 h post-injection^18^.

## Conclusions

DS is more practical than PS, with higher association with quantitative indexes on [^99m^Tc]Tc-DPD scan and SPECT/CT. H/CL, H/WB and SUVmax are effective quantitative indexes that have high correlations with visual grading. Particularly, SUVmax can be a reliable quantitative SPECT/CT index, with nearly perfect inter-observer agreement, high diagnostic power for determining visual grades, and potential association with ECV on cardiac MRI.

## Methods

### Patients and clinical data

Patients who were referred for [^99m^Tc]Tc-DPD whole-body scan and SPECT/CT from November 2018 to October 2020 in our institution were retrospectively enrolled. The patients were suspected as CA based on cardiologists’ examination, and those with previous history of pharmacological treatment for CA or cardiac transplantation were excluded. Patients’ clinical information and results of laboratory studies were retrieved from the health information system. Echocardiography and cardiac MRI that were performed within 1 year from [^99m^Tc]Tc-DPD imaging were also reviewed, excluding patients with definite is chemic cardiomyopathy.

The analyses in this study related to human participants were in accordance with the ethical standards of the Helsinki declaration in 1964 and its later amendments. The study design and waive of informed consent were approved by the Institutional Review Board of Seoul National University Hospital (H-2103-001-1200).

### [^99m^Tc]Tc-DPD scan and SPECT/CT

[^99m^Tc]Tc-DPD (740 MBq) was intravenously injected and the accurate injected radioactivity was calculated by measuring residual syringe activity after the injection. After 2–3 h, a whole-body scan of anterior/posterior views and SPECT/CT of the chest were sequentially performed. Images were obtained by using a single hybrid SPECT/CT scanner (Discovery NM/CT 670, GE Healthcare) equipped with low-energy general-purpose collimators. For a whole-body scan, table speed was set for total counts of 1.5–2.0 million. SPECT images were acquired using a step-and-shoot protocol, for 20 s per step with 3-degree intervals. SPECT images were reconstructed using an iterative algorithm (ordered subset expectation maximization) and the final matrix size was 128 × 128. Afterward, a helical CT was performed and CT images were reconstructed by an adaptive statistical iterative reconstruction technique (120 kVp; 40 mAs; matrix size 512 × 512; slice thickness 2.5 mm).

### Image analysis: visual assessment

Two nuclear medicine physicians (S.B. and J.G.) visually analyzed [^99m^Tc]Tc-DPD images independently. Planar images were graded according to the two grading systems; one proposed by Perugini et al. (Perugini grading system; PS) and the other by Dorbala et al. (Dorbala grading system; DS)^19,20^. The definition of grades in each grading system is summarized in Supplementary Table 3. In case of discrepancy between the two readers, consensus on grades were made for comparison with other indexes and clinical factors.

### Image analysis: quantitative indexes on planar image

**Figure 6:**
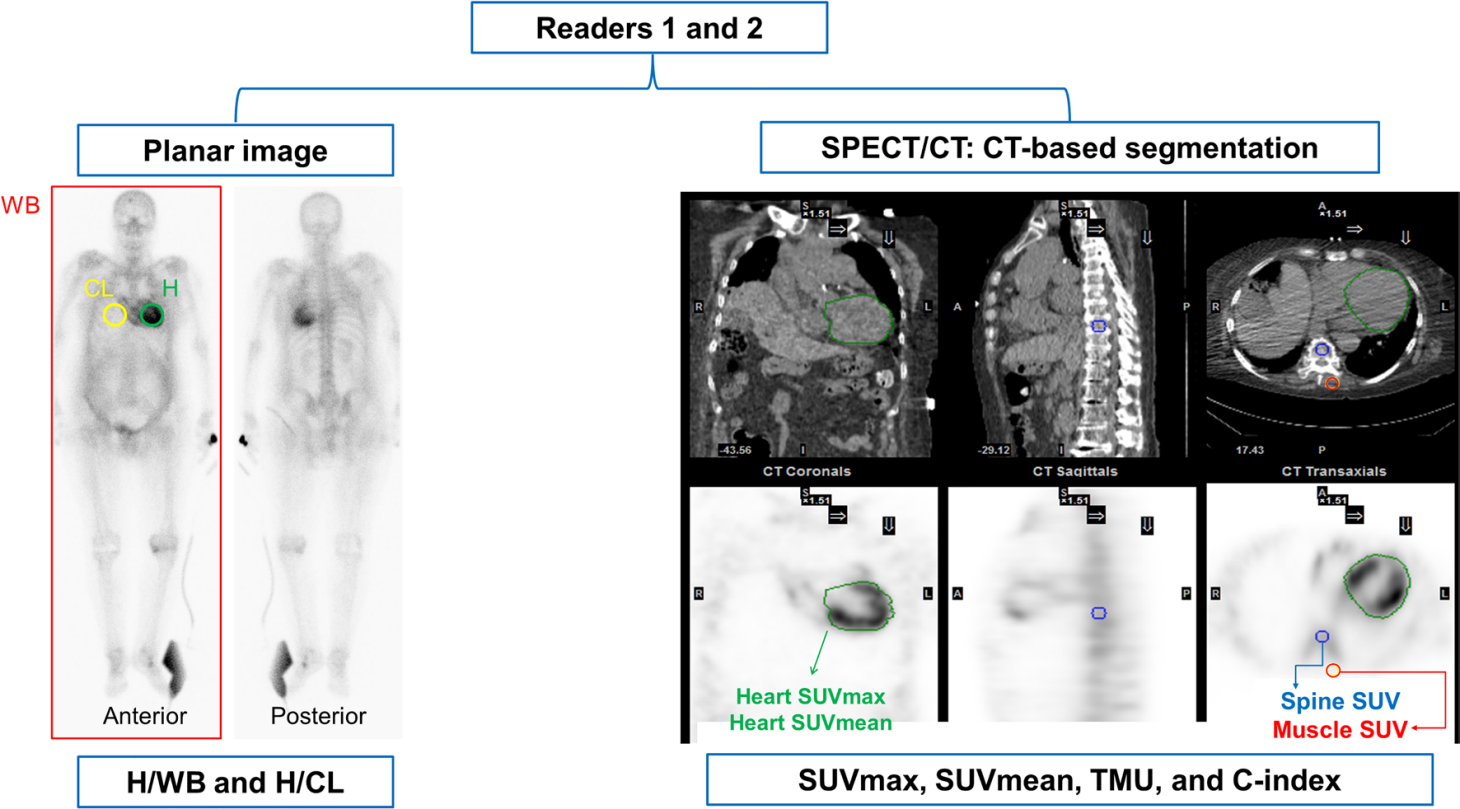
Study scheme and analysis methods of [^99m^Tc]Tc-DPD images. Quantitative indexes on planar image (H/WB and H/CL) and SPECT/CT (SUVmax, SUVmean, TMU, and C-index) were calculated and interobserver agreement was assessed between the two readers.

For quantitative analysis, heart-to-contralateral lung ratio (H/CL) and heart-to-whole body ratio (H/WB) were measured on planar images (Fig. 6). A circular region of interest (ROI) for the LV was drawn on the anterior view image, with excluding the sternum and spines. Another ROI of the same size and shape was drawn for the contralateral lung, and the ratio of mean counts of the ROIs was calculated as H/CL. To calculate H/WB, a rectangular ROI was drawn to encompass the whole body, and the total count of the ROI was measured. The ratio between the heart count and the whole body count was calculated, which was presumed to be the ratio of myocardial uptake to the total injected activity.

### Image analysis: quantitative indexes on SPECT/CT

On SPECT/CT images, standardized uptake value (SUV) was measured, and maximum SUV (SUVmax), mean SUV (SUVmean), total myocardial uptake (TMU), and cardiac uptake index (C-index) were obtained as quantitative indexes by using a vendor-supplied analysis system (Xeleris, GE Healthcare) (Fig. 6). ROIs for LV myocardium were manually drawn on transaxial CT images and were stacked to make a single volume of interest (VOI). A spherical VOI for the spine was drawn on the center of the thoracic vertebral body at the LV level, excluding abnormal lesions such as osteophytes. Another spherical VOI for the muscle was drawn on the left paravertebral muscle at the same level. From the LV VOI, SUVmax and SUVmean were measured, and TMU was calculated as [SUVmax × (volume of the LV VOI)]. C-index was calculated as [(SUVmax of LV) × (SUVmax of muscle)/(SUVmax of spine)], which means myocardial uptake normalized to bone uptake^7^. The C-index is assumed to show differences sensitively in Perugini grades 2 and 3, where the soft tissue uptake increases and bone uptake attenuates. The SPECT/CT indexes were acquired from the patients whose visual grades for planar image were ≥ 1. Among patients with visual grade 0, SPECT/CT indexes were measured in 50 randomly selected cases to reduce oversampling bias caused by many cases of visual grade 0. Two readers’ consensus values of quantitative indexes were also made for comparison with other indexes.

### Statistical analysis

Statistical analysis was performed using R software (Version 3.6.2, R Foundations for Statistical Computing, Vienna, Austria) with ggpubr (version 0.4.0), vcd (version 1.4–8), irr (version 0.84.1), and pROC (version 1.16.1) packages. The agreement of visual grades and SPECT/CT indexes by the two readers were tested using Cohen’s weighted κ value and two-way random, average-score intraclass correlation coefficients (ICCs). Bland–Altman plot and Wilcoxon signed-rank test were also applied to compare the SPECT/CT indexes. Kruskal–Wallis test was used to compare [^99m^Tc]Tc-DPD indexes between visual grades. As a post hoc analysis, Wilcoxon rank-sum test was utilized for pairwise comparison in each grade. Multiple comparison correction was performed based on Benjamini–Hochberg false discovery rate. Receiver-operating characteristics (ROC) analysis was used to determine optimal cutoff values and diagnostic efficacy of the quantitative indexes. [^99m^Tc]Tc-DPD image indexes were also tested for association with clinical manifestations; pathology, diastolic dysfunction indexes on echo (E/A and E/e’)^21,22^, and extracellular volume (ECV) on MRI^23^.

## Supplementary Information

Supplementary Information.

## Data Availability

The clinical information of the patients cannot be provided due to privacy issues. However, the analyzed data can be provided upon a request to the corresponding author.

## Abbreviations

CA: Cardiac amyloidosis
DPD: 3,3-Diphosphono-1,2-propanodicarboxylic acid
DS: Dorbala’s grade system
EMB: Endomyocardial biopsy
LV: Left ventricle
MRI: Magnetic resonance imaging
PS: Perugini’s grade system
PYP: Pyrophosphate
ROI: Region of interest
SPECT/CT: Single-photon emission computed tomography/computed tomography
SUV: Standardized uptake value
VOI: Volume of interest
