# Supplementary material for: Reliability and feasibility of visual grading systems and quantitative indexes on [99mTc]Tc-DPD imaging for cardiac amyloidosis
Source: Sci Rep. 2022 Oct 14;12:17271. doi: 10.1038/s41598-022-21603-8 (PMC9568548; doi:10.1038/s41598-022-21603-8)
Supplement: Supplementary file 1 — Supplementary Information. [file 41598_2022_21603_MOESM1_ESM.docx]

**Supplementary Information**

**Reliability and Feasibility of Visual Grading Systems and Quantitative Indexes on [^99m^Tc]Tc-DPD Imaging for Cardiac Amyloidosis**

**Supplementary Table 1.** Inter-observer agreement of DS and PS between the two readers

| Reader 1 | Reader 2 | | | |
| --- | --- | --- | --- | --- |
|  | 0 | 1 | 2 | 3 |
| DS |  |  |  |  |
| 0 | 132 | 0 | 0 | 0 |
| 1 | 3 | 4 | 0 | 0 |
| 2 | 0 | 0 | 2 | 0 |
| 3 | 0 | 0 | 1 | 10 |
| PS |  |  |  |  |
| 0 | 132 | 0 | 0 | 0 |
| 1 | 3 | 4 | 0 | 0 |
| 2 | 0 | 0 | 6 | 0 |
| 3 | 0 | 0 | 0 | 7 |

**Supplementary Table 2.** ICC values for [^99m^Tc]Tc-DPD indexes between the two readers (for those with DS ≥ 1)

| Modality | Indexes | ICC | 95% CI |
| --- | --- | --- | --- |
| Planar | H/WB | 0.918 | 0.403–0.977 |
|  | H/CL | 0.901 | 0.706–0.964 |
| SPECT/CT | SUVmax | 1.000 | 1.000–1.000 |
|  | SUVmean | 0.999 | 0.995–1.000 |
|  | TMU | 0.997 | 0.984–0.999 |
|  | C-index | 0.958 | 0.810–0.987 |

ICC, intraclass correlation coefficient; CI, confidence interval

**Supplementary Table 3.** Descriptions on each scoring system

| Scoring | Descriptions |
| --- | --- |
| DS |  |
| 0 | no myocardial uptake and normal rib uptake |
| 1 | myocardial uptake is less than rib uptake |
| 2 | myocardial uptake is equal to rib uptake |
| 3 | myocardial uptake is greater than rib uptake with mild or absent rib uptake |
| PS |  |
| 0 | absent myocardial uptake and normal bone uptake |
| 1 | mild myocardial uptake that is inferior to bone uptake |
| 2 | moderate myocardial uptake accompanied by attenuated bone uptake |
| 3 | strong myocardial uptake with mild or absent bone uptake |

**Supplementary Fig. 1. Bland-Altman plot for the two readers’ measurements on quantitative indexes (for those with DS ≥ 1)**

Blue lines represent the mean difference between the two readers and red lines represent 1.96 × SD of the differences.


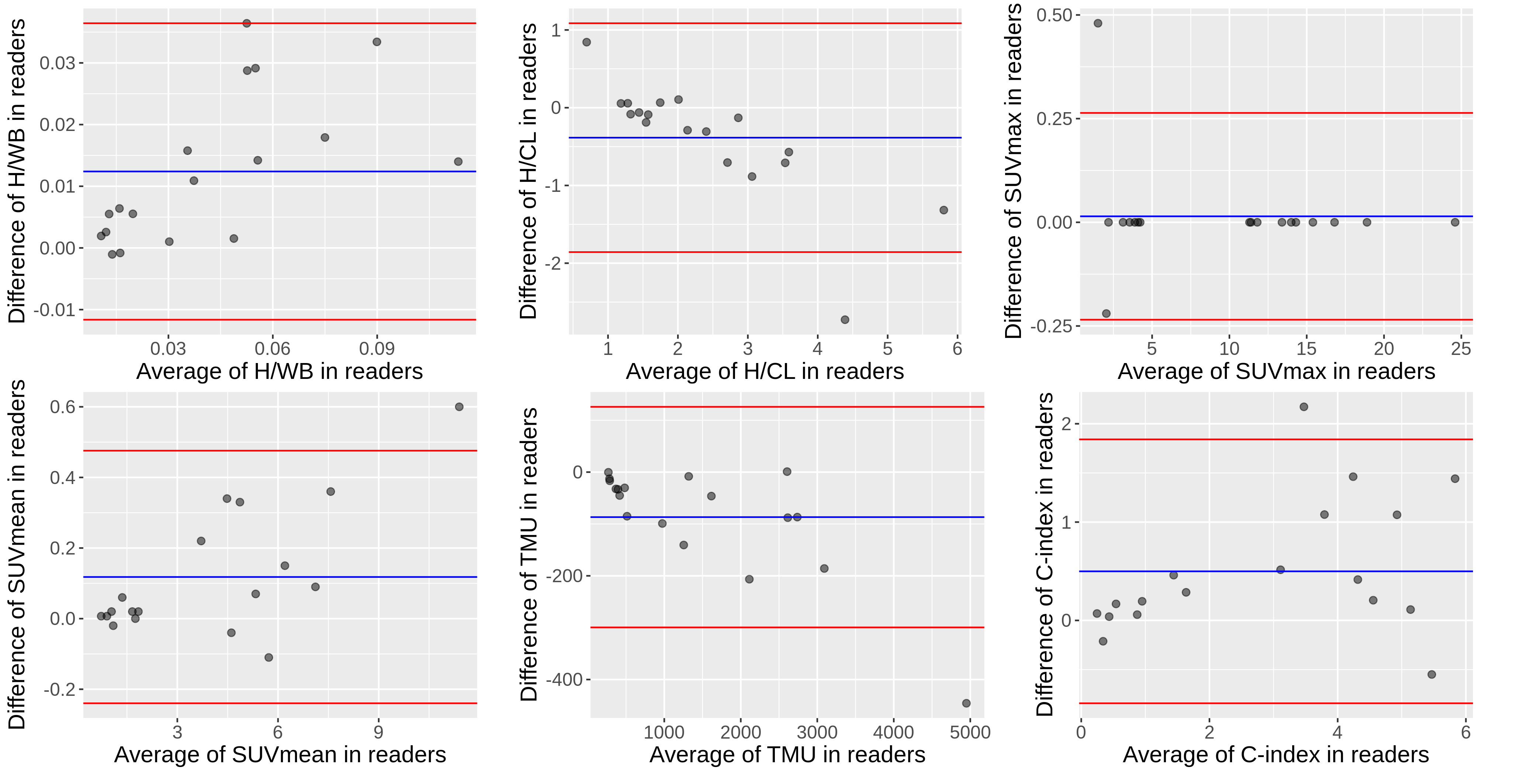


**Supplementary Fig. 2. Correlations of SPECT/CT indexes with planar indexes (for those with DS ≥ 1)**

Pearson’s correlation coefficients (R) and p-values are shown on top-left corners.


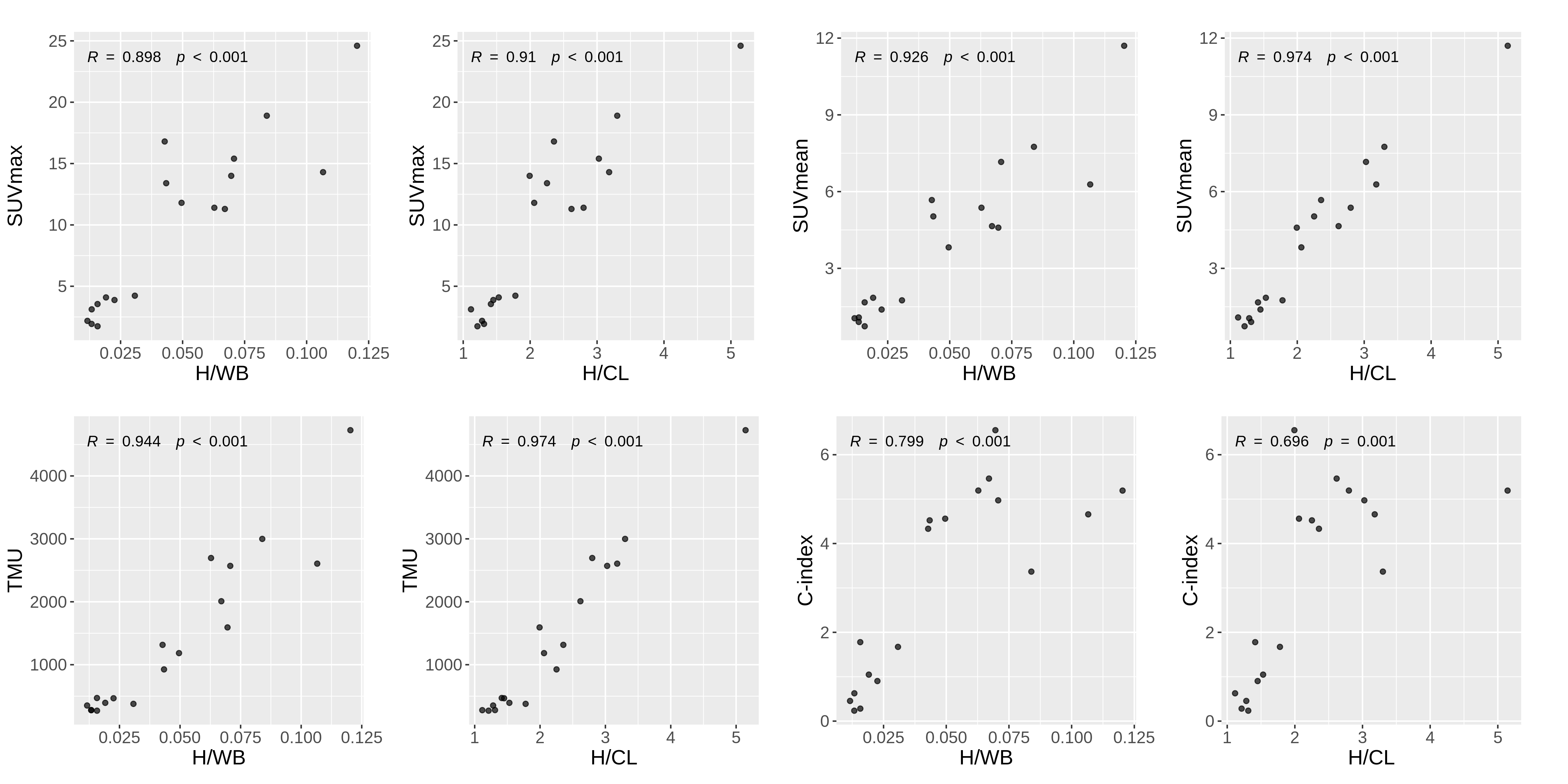


**a b**

**c d**

**Supplementary Fig. 3. Association of echocardiographic parameters with visual score and SUVmax**

**(a)** E/A and E/e’ did not show significant difference between different DS groups.

**(b)** Scatter plots presenting correlation between the echocardiography indexes with SUVmax. A weak correlation was observed between E/A and SUVmax.


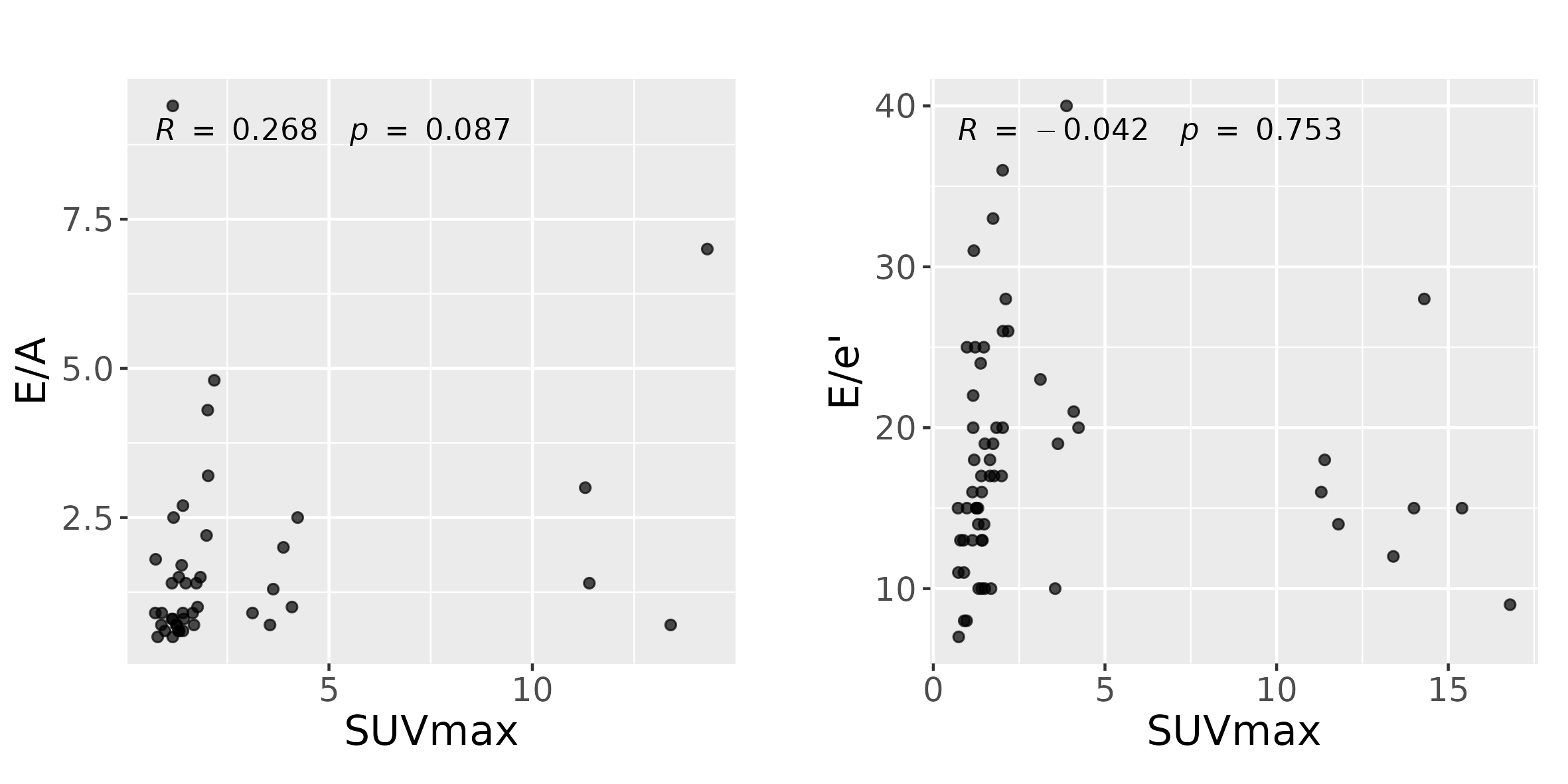


(**a) (b)**


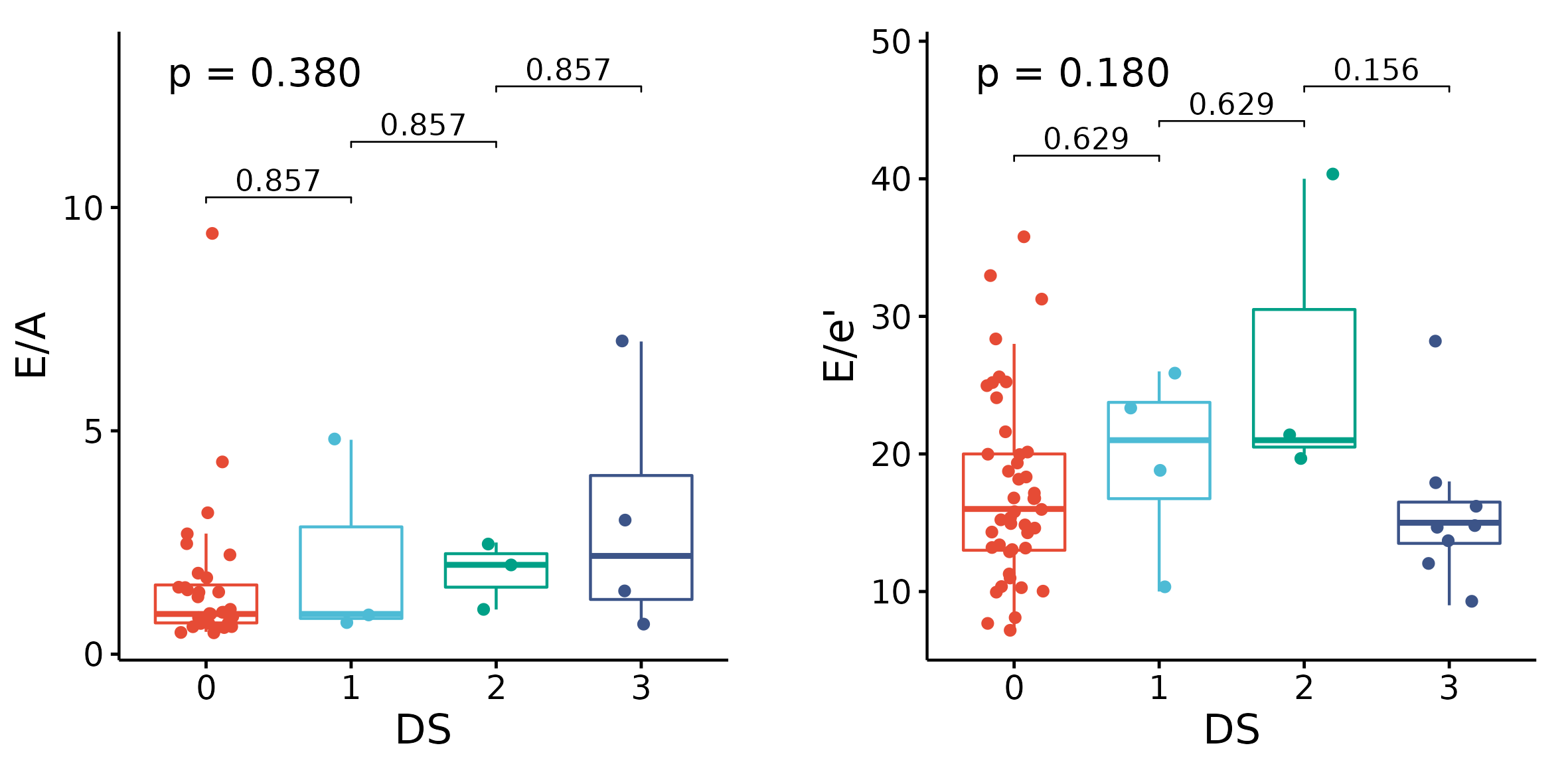


**Supplementary Fig. 4. Association of ECV with DS and SUVmax**

**(a)** ECV was significantly different between DS 0 and 3 (*P* = 0.015), although the difference was not significant between DS 0/1, and between 1/2, due to small sample sizes.

**(b)** Scatter plots presenting correlation between ECV and SUVmax. ECV showed a significant correlation with SUVmax (*P* < 0.001).


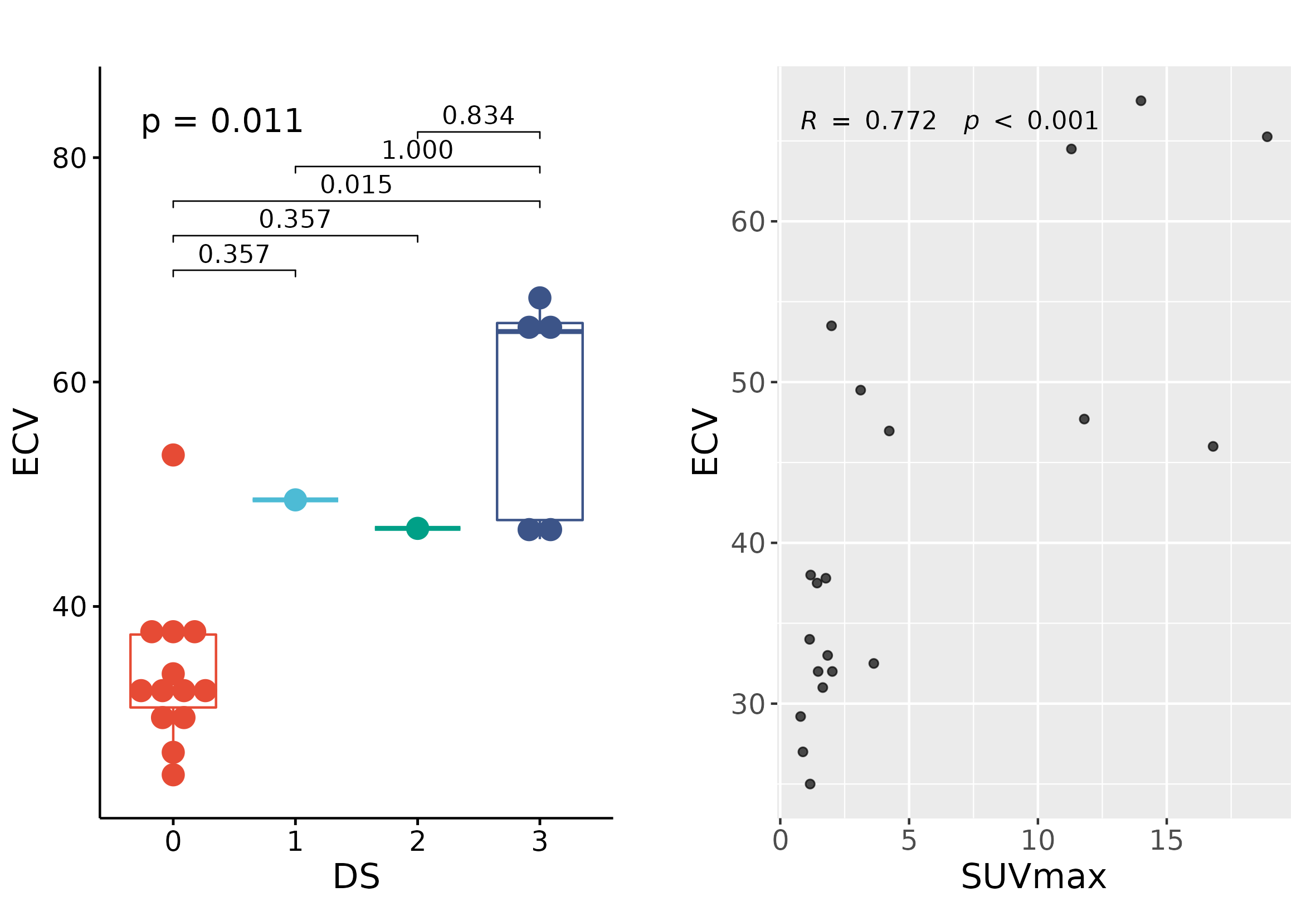


**(a)** (**b)**
